# Perfusion Characteristics of Hepatocellular Carcinoma at Contrast-enhanced Ultrasound: Influence of the Cellular differentiation, the Tumor Size and the Underlying Hepatic Condition

**DOI:** 10.1038/s41598-018-23007-z

**Published:** 2018-03-16

**Authors:** Dan Yang, Rui Li, Xiao-Hang Zhang, Chun-Lin Tang, Kuan-Sheng Ma, De-Yu Guo, Xiao-Chu Yan

**Affiliations:** 1 Department Ultrasound, Southwest Hospital Affiliated to Third Military Medical University, Chongqing, China; 2 Department Ultrasound, the Third Affiliated Hospital of Chongqing Medical University, Chongqing, China; 3 Department Hepato-biliary-Pancreatic Surgery, Southwest Hospital Affiliated to Third Military Medical University, Chongqing, China; 4 Department Pathology, Southwest Hospital Affiliated to Third Military Medical University, Chongqing, China

## Abstract

This study aimed to analyze the influence of the cellular differentiation, the tumor size and the underlying hepatic condition on the enhancement pattern of hepatocellular carcinoma (HCC) on contrast-enhanced ultrasound (CEUS). 276 patients with single lesion ≤ 5 cm who underwent CEUS exam and were pathologically confirmed as HCC were retrospectively enrolled. Enhancement patterns, washout patterns, wash-in time and washout time were observed and recorded. During the arterial phase, more poorly differentiated HCCs (42.5%) and lesions > 3 cm (35.2%) performed inhomogeneous enhancement (p < 0.05). More well differentiated HCCs (63.4%) performed late washout or no washout while compared with moderately (37.8%) or poorly (24.1%) differentiated HCCs (p < 0.05). Poorly differentiated HCCs showed the shortest washout time (83.0 ± 39.8 s), moderately differentiated HCCs showed the moderate washout time (100.4 ± 52.1 s), and well differentiated HCCs showed the longest washout time (132.3 ± 54.2 s) (p < 0.05). Lesions > 3 cm (97.2 ± 51.3 s) washed out more rapidly than lesions ≤ 3 cm (113.9 ± 53.5 s) (p < 0.05). The dynamic enhancement procedure of HCC was influenced by the cellular differentiation and the tumor size. While, hepatic background showed no influence on the dynamic enhancement of HCC.

## Introduction

Hepatocellular carcinoma (HCC) is the fifth most common cancer worldwide, and the third most common cause of cancer-related deaths worldwide^1^. Every year, there are more than 700000 cases diagnosed worldwide. So far, dynamic imaging techniques, namely contrasted enhanced ultrasound (CEUS), contrasted enhanced computed tomography (CT), magnetic resonance imaging (MRI), are the most common noninvasive diagnostic methods for HCC. The reported sensitivity and specificity of the diagnosis of HCC were 61.7–91.4% and 96.6–100% for CT or MRI, while 51.7–88%and 93.1–95.6% for CEUS^2–5^. CEUS possesses comparative capacity for diagnosing HCC while compared with CECT and MRI^6^. With the development of ultrasound contrast agent and dynamic real-time imaging techniques, CEUS has widely used in clinical practice and accepted in some national and international guidelines on HCC^7–9^.

The key feature for the diagnosis of HCC is hyper-enhancement in the arterial phase followed by wash out in the portal and/or late phase. However, studies revealed that the enhancement patterns of HCC on CEUS varied in some cases and the factors affecting these enhancement patterns are not well understood. Some investigators demonstrated that well differentiated HCC shows late washout or no washout, while wash out is observed more frequently in HCC with poorer grades of differentiation in portal or delayed phase^10–12^. Nevertheless, the analysis was performed between well differentiated HCC and less differentiated HCC in the previously mentioned studies, less differentiated HCC included both moderately and poorly differentiated HCC. The moderately differentiated and poorly differentiated HCC were not divided and the comparison between them not performed^10–12^. Some researchers reported that wash out is observed in about half the cases of small HCC but less in very small nodules (20–30% in those 1–2 cm, 40–60% in those 2–3 cm)^5,13^. The effect tumor size on wash out needs further investigation. In addition, whether the background of liver has an effect on the dynamic enhancement progress of HCC remains unknown.

The aims of this study were: (1) to analyze the time of wash-in and wash-out among different differentiated tumors retrospectively, especially between moderately and poorly differentiated HCC; (2) to investigate the influence of the tumor size on the enhancement of HCC at CEUS; (3) to clarify whether the background of liver has influence upon the enhancement pattern of HCC at CEUS.

## Materials and Methods

### Patients population

According to the guidelines from Declaration of Helsinki, institutional review board approval was achieved from the ethics committee of Southwest hospital. The need for informed consent of each patient was waived in this retrospective study. Between January 2005 and June 2015, 4370 patients who had CEUS examination and confirmed pathological diagnosis after surgical resection or ultrasound guided biopsy were retrospectively identified through a review of hospital database and records of the Department of Pathology Non-HCC pathological diagnosis was established in 1444 patients. Thus, we got 2926 cases of HCC. Clinical information (gender, age, etiology of cirrhosis, serum AFP value) was retrospectively collected from our hospital clinical information system. We performed a per-patient analysis. The inclusion criteria for the present study were as follows:

1. Patients with single solid nodule ≤ 5 cm;
2. Real-time CEUS for characterization of liver lesion was performed within less than a month before resection or biopsy;
3. The final diagnosis of HCC had to be verified pathologically through evaluation of surgical specimen or biopsy.

Exclusion criteria were:

1. Pathological diagnosis of hepatic lesion was non-HCC;
2. Tumor size > 5 cm which were not considered as fulfilling the Milan criteria for HCC and large tumors often have intra-tumoural necrosis that unpredictably influences the contrast pattern on CEUS;
3. Nodule number ≥ 2 because CEUS could not scan multiple nodules simultaneously after one injection of contrast agent if the nodules are not at the same scan plane and it is difficult to correspond the pathology of each tumor to the imaging of US in patients with multiple hepatic lesions;
4. Patients with thrombosis portal vein or hepatic vein which may influence the hepatic dynamic circulation;
5. Systemic chemotherapy or targeted treatment prior to the CEUS.

**Figure 1:**
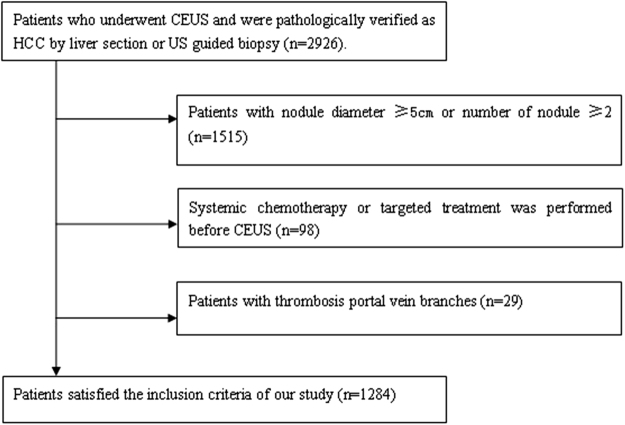
Flowchart of the inclusion and exclusion criteria of 2926 patients of hepatocellular carcinoma.

According to the inclusion and exclusion criteria mentioned above, 1284 patients with HCC satisfied the inclusion criteria of the study (Fig. 1) including 96 cases of well differentiated HCC, 1108 cases of moderately differentiated HCC and 80 cases of poorly differentiated HCC. The number of moderately differentiated HCC were too large to compare with that of well differentiated and poorly differentiated HCC statistically. One hundred cases were randomly selected from 1108 cases of moderately differentiated HCC at random, by creating the sequence numbers in the SPSS 13.0 software package (SPSS Inc,Chicago, IL). Finally, a total 276 cases of HCC (96 cases of well differentiated,100 cases of moderately differentiated and 80 cases of poorly differentiated) were included in this study.

### Image acquisition

Each patient receiving the CEUS examination was fasting for at least 8 h. US scanning was performed by two experienced physicians (R L, and XHZ) with more than 16 years of experience of liver ultrasound examination. An Acuson Sequoia 512 ultrasound unit (Siemens Medical Solutions, Santa Clara, Calif) with a multi-frequency transducer and contrast pulse sequencing (CPS) contrast-specific software was applied in conventional US scanning and CEUS. The characteristics of the lesion at gray-scale US, including location, size, echogenicity, shape and color Doppler, were recorded. Dual imaging mode was used to show the same scanning plane of gray scale US and CEUS simultaneously. SonoVue (Bracco Imaging B.V, Geneva, Switzerland) composed of phospholipids-stabilized microbubbles filled with sulphur hexafluoride gas was used as ultrasound agent throughout the study period. Contrast agent was administered as a bolus injection (2.4 ml during 3 seconds) followed by a 5 ml saline flush during 3 seconds with relatively constant speed. Manual injection of contrast agent was used by experienced nurses and a watch used to determine time. Real-time contrast imaging setting was used with a low mechanical index of <0.2 to avoid the bubbles disruption. And hepatic lesion was scanned continuously for up to 4 min after SonoVue injection. All images of CUES were digitally stored.

### Histological examination

The histological diagnosis was the gold standard of diagnosis. All cases obtained final diagnosis histologically by analyzing biopsy specimen (38 cases) or surgical section (238 cases). US-guided biopsy was done at both within the nodule and the adjacent liver tissue by18 gauge trenchant needle (Bard Peripherals Vascular Inc, Tempe, Arizona 85281, USA). The histological diagnosis of HCC was made according the International Working Party criteria^14^. The differentiation of tumor cells was graded as well differentiated, moderately differentiated, and poorly differentiated, primarily based on Edmondson and Steiner^15^. Liver sections or biopsy specimens were formalin-fixed, stained with haematoxylin and eosin (HE) followed by immunohistochemical staining. Final pathological diagnosis was made in consensus by two pathological experts with over 20 years experience of liver pathology (XCY and DYG) who were unaware to of the clinical information and CEUS findings. In this study, hepatic background was divided into cirrhosis and non-cirrhosis on the basis of pathology. Liver cirrhosis was histoligically proven through evaluation of hepatic parenchyma specimen after surgical or biopsy.

### CUES image analysis

Images were analyzed by two physicians with over sixteen years experience in abdominal diseases diagnosis (R L and XHZ) who were blind to the pathological diagnosis and the findings of CT or MRI. Contrast enhancement analysis was made in consensus between the two physicians. The whole enhancement procedure was studied, consisting of the arterial phase (0–30 s from the SonoVue bolus injection), portal phase (31–120 s after the injection), and delayed phase (121–240 s after the injection) according to EFSUMB recommendations^6^. The echogenicity of the lesion was noted as hyperechoic, isoechoic, hypoechoic, and mixed echo with respect to the surrounding liver tissue at baseline US. Contrast wash-in is considered as hyperechoic in comparison to the surrounding liver and the enhancement area is at least more than half of the lesion cross scanning section. The initial enhancement time and time to peak enhancement were registered separately.The initial enhancement time was defined as the time when contrast agent begins to emerge in the nodule after injection of contrast agent. The enhancement patterns of HCC at arterial phase were classified as follows:

1. Homogeneous enhancement—the whole nodule shows hyperechoic homogeneously compared with the surrounding liver parenchyma.
2. Inhomogeneous enhancement— when the lesion displays mixed hyper-enhancement at the periphery and the central part of the lesion, enhancement area involves more than half of the lesion.
3. Peripheral enhancement— irregular ring-like hyper-enhancement at the peripheral part of the lesion while sparse filiform and punctiform internal enhancement.
4. Iso-enhancement—enhancement degree of the lesion is similar to the surrounding liver parenchyma.
5. Hypo-enhancement—the lesion enhances in the less degree than that of surrounding liver tissue.
6. Non-enhancement—there is no enhancement (microbubbles do not appear) at both the periphery and the central part of the lesion.

Washout is defined as hyper-enhancement (including homogeneous or inhomogeneous enhancement) in arterial phase followed by hypoenhancement in the portal or/and the late phase. If the lesion shows inhomogeneous hyper-enhancement in the arterial phase, observation of washout is focused on the area of hyper-enhancement in the arterial phase. The lesion without hyper-enhancement in arterial phase is not defined as washout. The time of the nodule beginning to display washout was recorded. And the time of the nodule beginning to display washout was defined as the time when the nodule began to show hypoenhancement (hypo-echogenicity) compared with the surrounding hepatic parenchyma. The hepatic lesion showing similar degree or higher degree of enhancement in the delayed phase when compared with the adjacent liver parenchyma preceded by hyper-enhancement in the arterial phase is defined as no washout. According to the first appearance of hyoenhancement, washout patterns were classified as washout in the arterial phase, washout in the portal phase, washout in the delayed phase and no washout.

### Statistical analysis

Comparison of enhancement patterns of HCC with different histological grades, different tumor sizes and different hepatic backgrounds was done by using the chi-square test or Fisher exact method. Wash-in time, time to peak and wash-out time were expressed as mean ± standard deviation. Differences of cellular differentiation, tumor size and background of liver on wash-in time, time to peak and wash-out time were analyzed by independent-samples t test or one way of analysis of variance. The statistical evaluation was performed using the SPSS 13.0 software package (SPSS Inc, Chicago, IL). A P value less than 0.05 was considered as statistically significant for each test.

## Results

### Characteristics of patients

The clinical characteristics of the 276 cases of HCC are presented in Table 1. Mean age of the 276 patients was 51.1 ± 11.4 year(median: 50 year, range: 23–84 year) and 240 patients were male (86.9%). One hundred and sixty nine patients (61.2%) had cirrhosis. The etiology of cirrhosis was viral hepatitis B infection in 120 patients (71.0%), hepatitis B infection and alcohol misuse in 41patients (24.3%), alcohol misuse in 7 patients and viral hepatitis C infection in one patient. The remaining 107 (38.8%) patients did not have cirrhosis. Among them, sixty one patients had chronic hepatitis B without cirrhosis (57.0%). Twenty six patients (24.3%) had chronic hepatitis B and alcohol misuse but without cirrhosis. Fifteen patients had chronic alcoholic liver disease (14.0%). Five patients did not have any evidence of chronic liver disease. No significant difference was observed between the tumor diameter and tumor cellular differentiation (p > 0.05). The mean size of the lesions was 3.2 ± 1.0 cm (range: 1.0–4.9 cm).

**Table 1:** The clinical characteristics and grouping information of 276 cases.

Variable	Number (%)	Mean ± SD	Median (range)
Age (year)		51.1 ± 11.4	50 (23–84)
Male/female	240/36		
Nodule size (cm)		3.2 ± 1.0	2.8 (1.0–5.0)
Hepatic underlying diseases			
Cirrhosis	169 (61.2)		
Chronic hepatitis B	61 (22.1)		
Chronic hepatitis B and alcoholic	26 (9.4)		
Chronic hepatitis C	0		
Alcoholic	15 (5.4)		
No evidence of chronic liver disease	5 (1.8)		
Etiology of cirrhosis			
Chronic hepatitis B	120 (71.0)		
Hepatitis B and alcohol	41 (24.3)		
Alcohol	7 (4.1)		
Hepatitis C	1 (0.6)		
Cellular differentiation			
Well differentiated	96 (34.8)		
Moderately differentiated	100 (36.2)		
Poorly differentiated	80 (29.0)		
Tumor diameter			
≤3 cm	120 (43.4)		
>3 cm	156 (56.5)		
Hepatic condition (n)			
Cirrhosis	169 (61.2)		
Non-cirrhosis	107 (38.8)		

### Enhancement patterns in arterial phase

Two hundred and seventy out of 270 (97.8%) lesions were hyperenhanced during the arterial phase as compared to the adjacent parenchyma, including 203 (75.2%) lesions with homogeneous enhancement and 67 (24.8%) lesions with inhomogeneous enhancement. As for the remaining 6 lesions, 4 lesions (1.5%) were hyoenhancement and 2 (0.7%) lesions were peripheral enhancement in the arterial phase. The relationship between enhancement patterns in arterial phase and tumor cellular differentiation, tumor diameter and hepatic condition are presented in Table 2. There was no difference between proportion of hyperenhancement in arterial phase and cellular differentiation, tumor diameter, or hepatic condition (p > 0.05) (Table 2). Significant difference was only observed between homogeneous enhancement lesions and inhomogeneous enhancement lesions of different cellular differentiation and tumor size (p < 0.05) (Table 2). More well differentiated HCCs (84.4%) and moderately differentiated HCCs (77.0%) manifested homogeneous enhancement as compared with poorly differentiated HCCs (56.3%) (p < 0.05), while higher percentage of poorly differentiated HCCs (42.5%) enhanced inhomogeneously in arterial phase when compared with well differentiated HCCs (12.5%) and moderately differentiated HCCs (21.0%) (p < 0.05). The analysis between well differentiated HCCs and moderately differentiated HCCs showed no significant difference (p > 0.05). There was no significant difference between enhancement patterns in the arterial phase and hepatic condition, namely cirrhotic or non-cirrhotic liver. (Table 2).

**Table 2:** The enhancement patterns in arterial phase in terms of tumor cellular differentiation, tumor diameter and hepatic condition.

	Total	Hyperenhancement^1^	Homogeneous enhancement	Inhomogeneous enhancement	Peripheral enhancement	Hypo- enhancement
Cellular differentiation						
Well differentiated (n(%))	96	93(96.9)	81(84.4)	12(12.5)	0	3(3.1)
Moderately differentiation (n(%))	100	98(98.0)	77(77.0)	21 (21.0)	2(2.0)	0
Poorly differentiated(n(%))	80	79(98.8)	45(56.3)	34 (42.5)	0	1(1.3)
P value		0.711	0.000	0.000	0.170	0.184
Tumor diameter						
≤3 cm (n(%))	120	116(96.7)	104(86.7)	12(10.0)	0	4(3.3)
>3 cm (n(%))	156	154(98.7)	99(63.5)	55(35.2)	2(1.3)	0
P value		0.458	0.000	0.000	0.597	0.076
Hepatic condition						
Cirrhosis (n(%))	169	165(97.6)	124(73.4)	41(24.2)	0	4(2.4)
Non-cirrhosis(n (%))	107	105(98.1)	79(73.8)	26(24.3)	2(1.9)	0
P value		0.873	0.933	0.994	0.146	0.160

^1^Hyperenhancement involves homogeneous enhancement and inhomogeneous enhancement.

### Washout patterns

The relationship between washout patterns and cellular differentiation, tumor size and hepatic condition were presented in Table 3. Two hundred and thirty seven lesions (87.8%) with hyperenhancement (homogeneous or inhomogeneous) in arterial phase became hyoechogenicity compared with surrounding liver tissue in portal or/and delayed phase. The percentage of washout in the portal phase, washout in the delayed phase or no washout was significantly different among different cellular differentiation (p < 0.05) (Table 3). Further comparison revealed that a significantly higher percentage of moderately differentiated HCCs (61.2%) or poorly differentiated HCCs (74.4%) showed washout in portal phase than that of well differentiated HCCs (36.6%) (p < 0.05). And more well differentiated HCCs showed washout in delayed phase or no washout (41.9%, 21.5%, respectively) when compared with moderately differentiated HCCs (27.6%, 10.2%,respectively) or poorly differentiated HCCs (20.3%, 3.8%, respectively) (p < 0.05). The proportions of washout in portal phase, washout in late phase, and no washout were not significantly different between moderately and poorly differentiated HCCs (p > 0.05). No significance was found between washout patterns and tumor diameter or underling hepatic condition (p > 0.05) (Table 3).

**Table 3:** Various washout patterns in terms of tumor cellular differentiation, tumor diameter and hepatic condition.

	Total	Washout in arterial phase	Washout in portal phase	Washout in delayed phase	Without washout
Cellular differentiation					
Well differentiated (n(%))	96	0	34(36.6)	39(41.9)	20(21.5)
Moderately differentiation (n(%))	100	1(1.0)	60(61.2)	27(27.6)	10(10.2)
Poorly differentiated (n(%))	80	1(1.3)	59(74.7)	16(20.3)	3(3.8)
P value		0.579	0.000	0.006	0.001
Tumor diameter					
≤3 cm (n(%))	120	0	65(56.0)	40(34.5)	11(9.5)
>3 cm (n(%))	156	2(1.3)	88(57.1)	42(27.3)	22(14.3)
P value		0.508	0.856	0.202	0.233
Hepatic condition					
Cirrhosis (n(%))	169	1(0.6)	93(53.4)	51(30.9)	20(12.1)
Non-cirrhosis (n(%))	107	1(1.0)	60(57.1)	31(29.5)	13(12.4)
P value		1.000	0.900	0.809	0.949

### Wash-in and wash-out time

**Figure 2:**
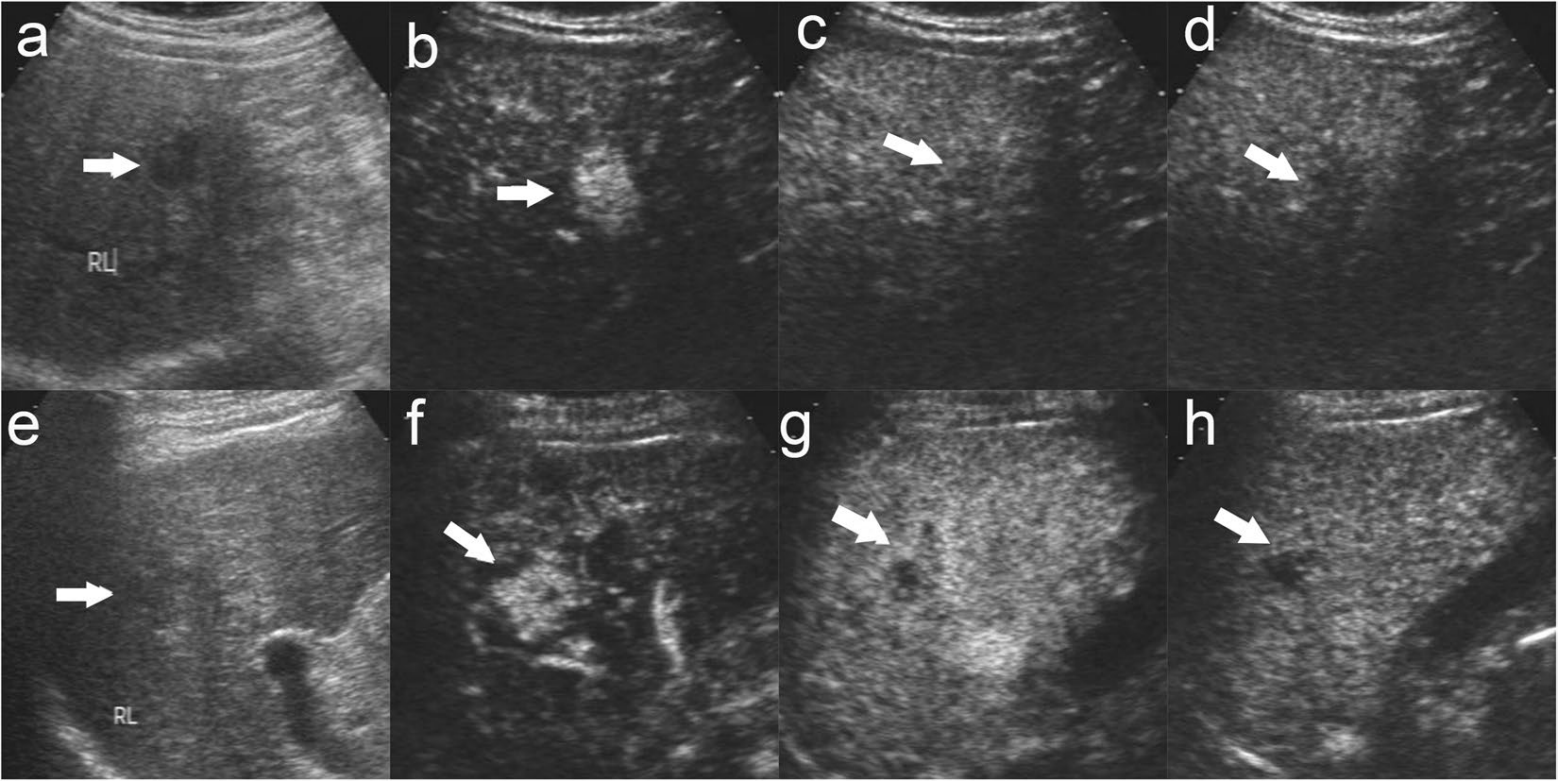
Enhancement features of moderately and poorly differentiated HCC. There was a well defined, hypoechoic nodule of 1.8 cm in the right lobe liver in a 59 year male patient (**a**). The nodule displays global hyperenhancement in the arterial phase ((**b**), 21 s after contrast agent injection) followed by slightly washout in the portal phase ((**c**), 120 s after contrast agent injection) and in the late phase ((**d**), 188 s after contrast agent injection). The patient was diagnosed moderately differentiated HCC with cirrhosis by us-guided biopsy. There was a well defined margin, slightly hypoechoic nodule of 2.0 cm in the right lobe liver in a 61 year male patient (**e**). The nodule displays heterogeneous hyperenhancemen in the arterial phase ((**f**), 21 s after contrast agent injection) followed by quick washout in the portal phase ((**g**), 64 s after contrast agent injection) and marked washout in the late phase ((**h**), 227 s after contrast agent injection). The patient was diagnosed poorly differentiated HCC with cirrhosis by us-guided biopsy. The lesion was indicated by white arrow.

**Figure 3:**
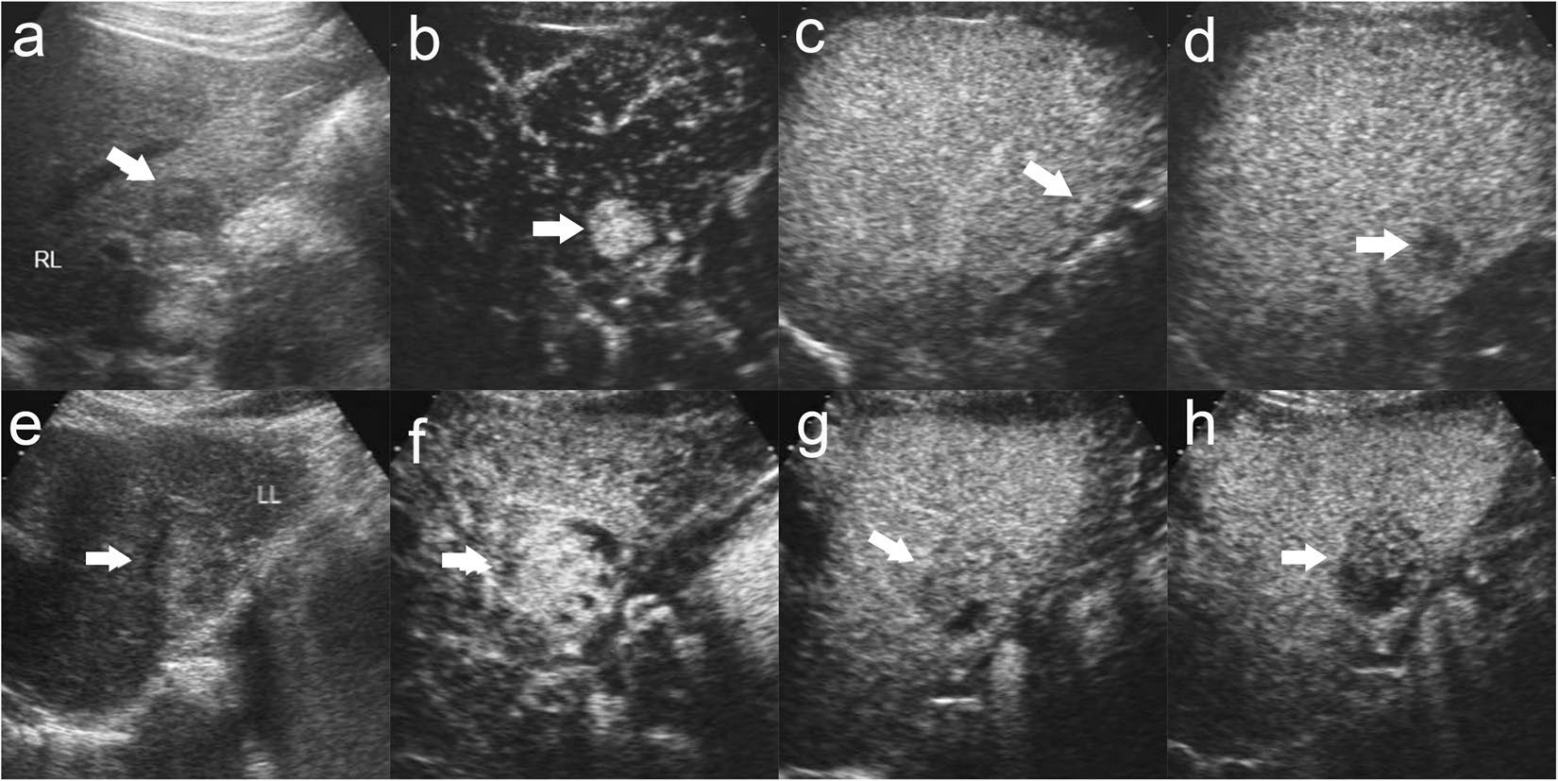
Influence of tumor size on Enhancement features of HCC. There was a well defined, hypoechoic nodule of 2.2 cm in the right liver lobe in a 38 year male patient (**a**). The nodule displays homogeneous hyperenhancement in the arterial phase ((**b**), 11 s after contrast agent injection). The nodule shows isoenhancement in the portal phase ((**c**), 87 s after contrast agent injection). The nodule shows washout in the late phase ((**d**), 208 s after contrast agent injection). The patient was diagnosed moderately differentiated HCC without cirrhosis by surgery. There was a well defined margin, slightly hypoechoic nodule of 3.5 cm in size with hypo-echoic halo in baseline us in the left lobe liver in a 64 year male patient (**e**). The nodule displays heterogeneous hyperenhancemen in the arterial phase ((**f**), 22 s after contrast agent injection) followed by quick washout in the portal phase ((**g**), 52 s after contrast agent injection) and moderate washout in the late phase ((**h**), 159 s after contrast agent injection). The patient was diagnosed moderately differentiated HCC without cirrhosis by surgery. The lesion was indicated by white arrow.

**Figure 4:**
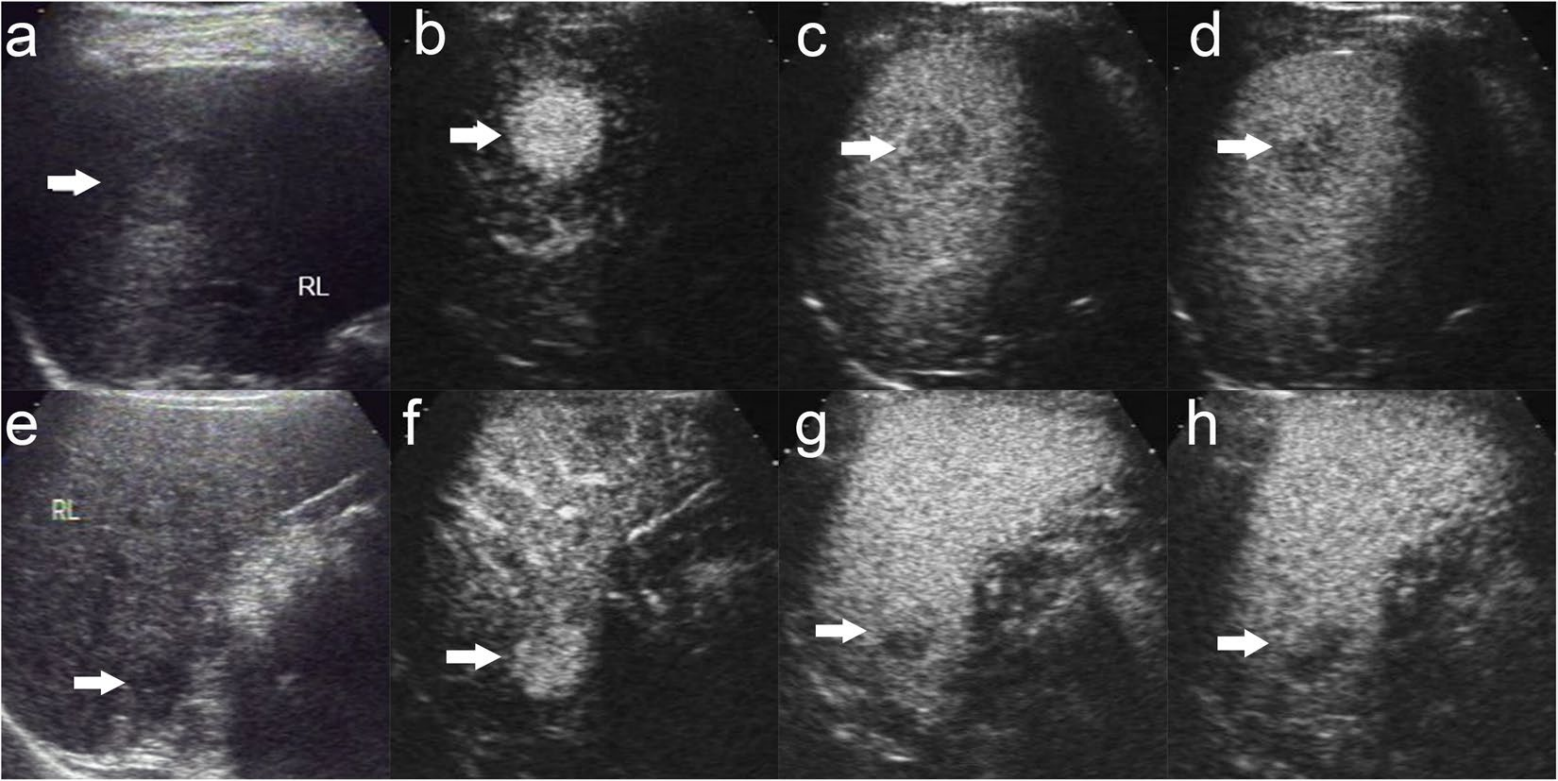
Enhancement features of HCC with cirrhosis and non-cirrhosis. There was a well defined, hypoechoic nodule of 2.8 cm with hypo-echoic halo in baseline us in the right lobe liver in a 56 year male patient (**a**). The nodule shows global hyperenhancemen in the arterial phase ((**b**),14 s after contrast agent injection) followed by slight washout in the portal phase ((**c**), 85 s after contrast agent injection) and moderate washout in the late phase ((**d**), 135 s after contrast agent injection). The patient was diagnosed poorly differentiated HCC without cirrhosis by surgery. There was a vague margin, slightly hypoechoic nodule of 2.4 cm in the right lobe liver in a 46 year male patient (**e**). The nodule displays heterogeneous hyperenhancemen in the arterial phase ((**f**), 23 s after contrast agent injection) followed by washout in the portal phase ((**g**), 80 s after contrast agent injection) and marked washout in the late phase ((**h**), 185 s after contrast agent injection). The patient was diagnosed poorly differentiated HCC with cirrhosis by surgery. The lesion was indicated by white arrow.

The average wash-in time of the 272 lesions with hyperenhancement or peripheral enhancement in the arterial phase was 13.1 ± 5.1 s. The average washout time of 237 lesions with hyperenhancement in arterial phase followed by wash out in the procedure was 104.6 ± 58.4 s. Table 4 shows the relationship between dynamic enhancement time and tumor cellular differentiation, tumor size and underling hepatic condition. The initial wash-in time and time to peak showed no significant difference in terms of cellular differentiation, tumor size and underling hepatic condition (p > 0.05) (Table 4). Wash out time was significantly different between HCCs with different cellular differentiation. Poorly differentiated HCCs showed shortest washout time (83.0 ± 39.8 s), moderately differentiated HCCs showed the moderate washout time (100.4 ± 52.1 s), and well differentiated HCCs (132.3 ± 54.2 s) showed the longest washout time (p < 0.05) (Fig. 2). The same results were got among the patients with cirrhosis (well differentiated HCCs: 130.5 ± 52.1 s; moderately differentiated HCCs: 105.9 ± 52.5 s; poorly differentiated HCCs: 84.0 ± 41.7, l p < 0.05) or without cirrhosis (well differentiated HCCs:136.0 ± 59.1 s; moderately differentiated HCCs: 94.5 ± 51.6 s; poorly differentiated HCCs: 80.7 ± 36.4 s p < 0.05). Tumor diameter had significant correlation to washout time. Lesions > 3 cm displayed more rapid wash out (97.2 ± 51.3 s) than lesions ≤ 3 cm (113.9 ± 53.5 s) (p < 0.05) (Table 4) (Fig. 3). However, underlying hepatic condition did not exert an influence on the washout time of HCC on CEUS (p > 0.05) (Table 4) (Fig. 4).

**Table 4:** Time of CEUS enhancement in terms of tumor cellular differentiation, tumor diameter and hepatic condition.

	Initial time	Time to peak	Washout time
Cellular differentiation			
Well differentiated (n(%))	12.8 ± 4.9	19.1 ± 6.2	132.3 ± 54.2
Moderately differentiation (n(%))	12.2 ± 4.6	17.9 ± 5.8	100.4 ± 52.1
Poorly differentiated (n(%))	13.3 ± 6.6	20.1 ± 8.5	83.0 ± 39.8
P value	0.379	0.107	0.000
Tumor diameter			
≤3 cm (n(%))	12.8 ± 4.6	18.6 ± 5.7	113.9 ± 53.5
>3 cm (n(%))	12.7 ± 5.9	19.2 ± 7.6	97.2 ± 51.3
P value	0.908	0.437	0.015
Hepatic condition			
Cirrhosis (n(%))	12.9 ± 4.1	19.0 ± 5.1	106.5 ± 52.2
Non-cirrhosis (n(%))	13.4 ± 6.3	19.0 ± 8.9	101.6 ± 54.0
P value	0.400	0.996	0.485

## Discussion

CEUS, with purely intravascular contrast agent and contrast specific imaging techniques, was included in the diagnostic algorithm and recommended by EFSUMB in the diagnosis of focal liver lesions^16^. CEUS had the advantages of high sensitivity in observing hypervascularity in arterial phase and the real-time perfusion of the lesion. The natural history of the majority of HCC is multistep hepatocarcinogenesis^17^. In the malignant transition process, the supplying vessels undergo a serious changes, normal arteries and portal veins were decreasing while abnormal neoplastic arteries increasing^18^. To some extent, these changes correlated with enhancement patterns of HCC in arterial phase on CEUS. Hyper-enhancement in arterial phase was found in 97.8% of HCC lesions in the present study. The results were comparable to other groups, who found hypervascularization in 96.2%^19^, 95%^12^, 90%^20^of HCC lesions in early phase. However, Von Herbay *et al*.^21^ reported that 70.7% of HCC nodules showed arterial hypervascularization, while 29.3% showed non-hyperenhancement. This difference may be explaned by the differentiation of HCC nodules included. Von Herbay *et al*.included G4 HCCs (undifferentiated HCCs) in their study while our study and other researchers mentioned above did not. The histologial basis of hypoenhancement in arterial phase of HCCs was probably the decrease of artery supply. About 1.4% of HCC lesions displayed arterial hypoenhancement in our study which was similar to Nicolau’s^19^ study that 3.8% of HCC lesions showed arterial hypoenhancement. In the two studies, HCCs with hypoenhancement was mainly well differentiated HCCs (75% in our study and 100% in Nicolau’s study). These results differed from that of von Herbay’s^21^, in which 28% HCCs manifested non-enhancement (130 cases were enrolled in their study) and non-hyperenhancement occurred in all differentiated HCCs (24.3% in G1 cases, 35.1% in G2 cases, 21.6% in G3 cases, 16.2% in G4cases,and 2.7% in HCC/CCC cases). The possible reason could be the difference of US scan unit (Sonoline Elegra sonography unit in von Herbay’s study vs Acuson Sequoia 512 ultrasound unit in our and Nicolau’s study). Another reason could be no interpretation about non-hyperenhancement, which maybe includ iso-enhancement, hypo-enhancement, and peripheral enhancement. 1.5% of hypo-enhancement and 0.7% peripheral rim-like enhancement are in our study. Demonstrating that hypo-enhancement and peripheral rim-like enhancement are atypical for HCC. Hypoenhancement in delayed phase was found in 87.8% of HCC lesions in present study which was comparable to other studies by Jang^10^ (92.0%), Boozari^12^ (77.9%) and von Herbay^21^ (86.2%). However, Iavarone and colleagues found that the percentage of hypoenhancement in the late phase was 34%, and the lesions ≤ 2 cm accounted for 66.1% of all lesions with size between 0.9–5 cm (median 1.6 cm) in their study^11^. The average washout time of all lesions with hyperenhancement in arterial phase followed by wash out in portal or/and delayed phase was 104.6 ± 58.4 s, which was comparable to that reported by Iavarone *et al*. (121.2 ± 71.2 s), but was longer than Kong’s study (59.8 ± 20.5 s)^22^. The reason of the discrepance could be that Kong *et al*. included 95 recurrent lesions (accounted for 57.9% of all lesions) in their study.

With regard to the relationship between tumor cellular differentiation and washout patterns, well differentiated HCCs (namely G1-HCC in other literatures) showed more often a washout in delayed phase (41.9%) or no washout (21.5%) in present study. These results was similar to some published studies^10,12,23^. Jang *et al*.^10^ reported that 29% (4/14) of well differentiated HCCs showed washout after 3 min and 50% (7/14) of well differentiated HCCs showed no washout. However, Boozari *et al*.^12^ reported that 94.4% (17/18)of G1-HCCs showed persistent enhancement in late phase. The difference may be attributed to sample number and tumor size. In Boozari’s study, they only analyzed 18 G1 differentiated HCCs with 9 lesions < 2 cm in comparison to ours with more well differentiated HCC lesions (96 lesions in all) and more larger size lesions (80 lesions > 2 cm). Besides, the average time of emergence of washout in well differentiated HCCs was 132.3 ± 54.2 s, which was similar to Loria’s^24^ study (138.0 ± 114.5 s) and was significantly longer than moderately or poorly differentiated HCCs. The possible reason for late washout in well differentiated HCCs could be that the well differentiated HCC consisted of a trabecular pattern of cell cords and rich sinusoids that may lead to stagnation and slow clearing of microbubbles^25^. Our results demonstrated that HCCs with poorer grades of differentiation showed more often a washout in the portal phase than in well differentiated HCCs (p < 0.05). Washout patterns between moderately and poorly differentiated HCCs were not significantly different (p > 0.05). These results were comparable to other groups, who analyzed the difference between G1 differentiated HCC and G2-G3 differentiated or G2-G4 differentiated HCC^11,12,23,24^. However, statistically different results were observed by analyzing the time of initial washout in our study. Poorly differentiated HCCs (83.0 ± 39.8 s) showed the shortest washout time, moderately differentiated HCCs (100.4 ± 52.1 s) followed, and well differentiated HCCs (132.3 ± 54.2 s) the longest washout time. In our study, we observed that percentage of washout between moderately and poorly differentiated HCCs did not reveal statistical difference, whereas time of washout emergence between moderately and poorly differentiated HCCs was statistically different. This was an important finding and first reported up to now. If a nodule displays hyperenhancement in arterial phase followed by washout in early portal phase, suspicious of poorly differentiated HCC should be included in the differential diagnosis in patients with chronic liver disease. The possible explanation for early washout in poorly differentiated HCC lesions could be that this kind of HCC contained less sinusoids and were mainly nourished by hepatic artery^25^.

Tumor diameter plays a role in the prognostic process of HCC. In our study, there existed no relationship between tumor diameter and cellular differentiation. These findings were in agreement with von Herbay *et al*.^21^, but in contrast to Fan *et al*.^23^ who found that tumors with larger size were significantly less differentiated HCC. Both our present study and Nicolau *et al*.^19^ (by using a cut-off diameter of 2 cm) demonstrated that the hyperenhancement patterns and washout patterns were not related to tumor diameter. In contrast, von Herbay^21^ found that more lesions (92%) > 3 cm manifested hyoenhancement in late phase than lesions <3 cm (77%). The same result was got in G1 subgroup lesions (95% of lesions > 3 cm vs 64% lesions < 3 cm). However, their study included fewer lesions in comparison to ours (in our study: 120 lesions ≤ 3 cm vs 156 lesions > 3 cm; in Nicolau’s study: 36 lesions < 2 cm vs 68 lesions > 2 cm; in von Herbay’s study: 53 lesions < 3 cm vs 77 lesions > 3 cm), thus, our data may lead to a better comparison between the two groups statistically. In addition, a significant percentage of lesions > 3 cm enhanced inhomogeneously in our study. The result was comparable to Fan’s study^23^, with 64.6% of less differentiated HCCs, resembling to ours (65.2% of less differentiated HCCs), indicating more necrosis in large HCCs. Further more, time of initial hyoenhancement was significantly shorter in lesions > 3 cm in our study which included more moderately to poorly differentiated HCCs when compared with lesions ≤ 3 cm.

CEUS was widely used in the diagnosis of HCC in Europe and Asia. Though most HCC occur in cirrhotic liver, it may also happen in patients with chronic hepatitis. Therefore, it is of clinical importance to know whether underlying hepatic background affect the enhancement patterns of HCC on CEUS. Jang *et al*.^10^ found that the proportions of each washout time interval were not significantly different between the two groups (no fibrosis and serve fibrosis). In our study, underlying hepatic background did not show correlation with perfusion characteristics of HCC, including enhancement patterns in arterial phase, washout patterns, and time of first occurrence of washout. It was an important finding, because HCC could occur in the liver without cirrhosis. Therefore, the noninvasive criteria could be probably applied to the HCC cases without cirrhosis, especially in patients with chronic hepatitis.

There were several limitations in our study. First, the sensitivity and specificity were not calculated. The results of our study need to be validated in a prospective study in the future. Second, the US scan unit in our study (Acuson Sequoia 512) has a relatively higher mechanical index of CEUS mode than other unit which maybe lead to earlier disruption of the microbubbles. Third, only the lesions ≤ 5 cm were enrolled in our study. We did not know whether the conclusions we got were adaptable to the lesions > 5 cm, in which the necrosis was more easily discovered.

In conclusion, global arterial enhancement and late washout were manifested in more well differentiated HCC than moderately or poorly differentiated HCC. Poorly differentiated HCC showed shorter washout time than moderately differentiated HCC. Large HCC (>3 cm) displayed more heterogeneous enhancement in the arterial phase and faster washout when compared with smaller HCC (≤3 cm). Underlying hepatic background showed no influence on the dynamic enhancement of HCC on CEUS.

### Data availability statement

Our original data cannot be made available in the manuscript, the supplemental files, or a public repository because of ethical restriction of the ethics committee of Southwest hospital.
